# Vascular variant of Eagle syndrome: a review

**DOI:** 10.3389/fneur.2024.1463275

**Published:** 2024-10-08

**Authors:** Joy Tadjer, Yannick Béjot

**Affiliations:** Department of Neurology, Dijon Stroke Registry, University Hospital of Dijon, EA7460, University of Burgundy, Dijon, France

**Keywords:** Eagle syndrome, elongated styloid process, stylocarotid syndrome, stroke, laminar cortical necrosis

## Abstract

Eagle syndrome is defined as an elongated styloid process (ESP) that compresses nearby vasculo-nervous structures. The vascular variant of Eagle syndrome can lead to neurological symptoms including syncope, transient ischemic attack, or stroke; however, it has also been associated with other atypical presentations, making its diagnosis challenging. This review aimed to depict the characteristics of patients with the symptomatic vascular variant of Eagle syndrome. The literature search identified 56 reported cases of vascular variants of Eagle syndrome, with a mean age at onset of 51 years (range: 15–85 years), and the male-to-female ratio was 2:4. The ESP was bilateral in 63% of the cases, and the mean length was 48 mm (range: 31–77 mm). Vascular complications were mostly represented by internal jugular vein (IVJ) stenosis (*n* = 28), followed by internal carotid artery (ICA) dissection (*n* = 15). Additionally, eight cases of ICA thrombosis and two cases of severe chronic stenosis of the ICA > 90% were reported. Vascular complications may lead to cerebral ischemia due to either a thromboembolic mechanism or, less frequently, reduced blood flow. Laminar cortical necrosis, as a cerebral complication of ICA compression, was exceptionally described in one case, and such an atypical clinical presentation may be regarded as a diagnostic pitfall.

## Introduction

Eagle syndrome was first described in 1937 by Watt Eagle and is defined by the ossification of the stylohyoid ligament, which becomes abnormally long and may compress nearby vasculo-nervous structures, often presenting as cervicofacial pain. The length of the styloid process is typically 25–30 mm, and a length of >30 mm is considered elongated. The incidence of Eagle syndrome is approximately 4% in the general population, and only 4% of patients with Eagle syndrome exhibit symptoms (1). Another manifestation of Eagle syndrome is stylocarotid artery syndrome, a vascular variant of Eagle syndrome that can lead to neurological symptoms, including syncope, transient ischemic attack, or stroke (2). However, other atypical presentations have also been reported. This article aimed to review the literature to depict the characteristics of patients with the symptomatic vascular variant of Eagle syndrome.

## Literature review

A literature search was performed in February 2023 in the PubMed and Web of Science databases using the following keywords: (“Laminar cortical necrosis” OR “stroke” OR “brain vascular accident” OR “apoplexy”) AND (“Eagle syndrome” OR “stylohyoid syndrome” OR “styloid syndrome” OR “styloid-carotid artery syndrome” OR “elongated styloid process” OR “stylocarotid syndrome”), without time restriction.

A total of 31 and 35 articles were found in PubMed and the Web of Science, respectively. After removing duplicates, 36 articles remained. We excluded seven more articles because they were not relevant to our topic. The excluded articles were as follows: one article focused on a fracture of the styloid process found in a skeleton, another was about the prevention of vascular complications during neck surgery, four articles did not discuss any vascular complications, and the last article was a review of seizure-induced reversible brain MRI abnormalities in status epilepticus.

We analyzed the data from the remaining 29 articles, which are summarized in Table 1. The quality of the reported cases was assessed according to the criteria of the CARE checklist, which outlines the information to include when writing a case report (3). Quality was classified as good (when all key information was present in the article), moderate (when some non-major information was missing), and insufficient (when important information was missing). This classification was not applied to one article that presented aggregate data from a case series of patients (4). Of the remaining 28 articles, 22 were classified as good quality, 2 as moderate quality, and 4 as insufficient quality (3 of which were conference poster abstracts).

**Table 1:** Vascular variant cases of Eagle syndrome reported in the literature.

Authors	Age (years) and sex	Study quality	Laterality of Eagle syndrome	Diagnosis
Shindo et al. 2019 (20)	49, M	A	Right (31 mm)	Right ICA occlusion of the distal cervical segment and ipsilateral MCA occlusion of the M3 branch
Torikoshi et al. 2019 (6)	46, M	A	Bilateral (>40 mm)	Bilateral ICA dissection and acute infarction in the right frontal and parietal lobes
Ikenouchi et al. 2020 (7)	30, M	A	Bilateral (length unspecified)	Bilateral ICA dissection and acute cerebral infarct in the right hemisphere
Jelodar et al. 2018 (21)	40, M	A	Bilateral (length unspecified)	Thrombus formation in the wall of the CCA on both sides and bilateral ischemic stroke
Razak et al. 2014 (8)	41, M	A	Bilateral Right: 45 mm Left: 50 mm	Right ICA dissection with intraluminal thrombi and distal occlusion of the M1 segment of the right MCA with right frontal–parietal–temporal infarct
Subedi et al. 2017 (9)	47, F	A	Bilateral at 42 mm	Right ICA dissection and a hyperacute infarct in the right MCA territory involving the middle frontal gyrus
Bai et al. 2020 (4)	27 patients 15 M/12F Age 59.7 ± 11.3	N/A	Average of 37 mm	IJVS stenosis: left (*n* = 5), right (*n* = 6), and bilateral (*n* = 16)
Zhang et al. 2019 (5)	15, M	B	Left (length unspecified)	Left IJVS and left transverse-sigmoid sinus thrombosis
Smoot et al. 2017 (10)	60, M	A	Bilateral Right: 69 mm Left: 44 mm	Right ICA dissection and numerous microinfarcts in the right parietal cortex, the periventricular area, and the frontal cortex
Zammit et al. 2018 (11)	45, M	A	Bilateral Right: 40 mm Left: 42 mm	Bilateral ICA dissection, old left frontal lobe infarct, and acute right MCA territory infarct involving the inferior division of the M2 segment
Kazmierski et al. 2018 (28)	51, M	A	Bilateral Right: 47 mm Left: 48 mm	Ischemic stroke in the left hemisphere within the vascular territory between the MCA and ACA caused by compression and vasospasm of the left ICA and an incomplete circle of Willis (absence of the A1 segment of the right AComm and hypoplasia of the P1 segment of the right PComm)
Budinčević et al. 2018 (19)	85, M	A	Right (31.3 mm) with impingement on the right ECA	Occlusion of the right ICA due to compression by a giant pseudoaneurysm of the right external ECA and cerebral infarct in the right parietooccipital region
Ohara et al. 2012 (22)	43, M	A	Bilateral (37 mm)	Right ICA occlusion and acute infarction in the right insular cortex and temporal lobe
Tanti et al. 2021 (12)	Young student (age unspecified), M	B	Bilateral Right: 74 mm Left: 70 mm	Right ICA dissection and M1 thrombus
Sveinsson et al. 2013 (2)	Case 1: 38, M Case 2: 41, F	A	Case 1: left Case 2: right	Case 1: left ICA dissection and left M1 thrombus Case 2: dissection of the right ICA with a small pseudoaneurysm. No infarct
Hebant et al. 2017 (13)	57, M	A	Bilateral Right: 31 mm Left: 33 mm	Left ICA dissection and left superficial MCA territory infarction and occlusion of the M3 segment
Qureshi et al. 2019 (1)	42, F	A	Bilateral Right: 60 mm Left: 45 mm	Intimal tear and dissection of the left ICA bulb, occlusion of the left MCA (M1 portion), and left basal ganglia ischemic stroke
Kesav et al. 2020 (14)	49, M	A	Bilateral Right: 61 mm Left: 57 mm	Dissection flap in the right distal cervical ICA, as well as occlusion of the left cervical ICA immediately after its origin and occlusion of the left MCA M1 segment
Ramadan et al. 2021 (23)	47, M	A	Bilateral (40 mm on the right)	Thrombus focal narrowing of the right ICA and right MCA territory infarction
Keshelava et al. 2021 (24)	67, M	A	Bilateral (length unspecified)	Left ICA stenosis (60%), right ICA tortuosity, and occlusion of the right CCA, with a minor ischemic stroke in the right centrum semiovale; no stroke foci were detected on the left side
Kavi and Lahiri 2016 (29)	70, M	C	Left (length unspecified)	Compression of the left extracranial ICA, with restricted cortical diffusion in left ACA and MCA territories, sparing the white matter
Keshelava et al. 2012 (25)	37, M	A	No ESP. Right ICA suffered from manual compression during sleep	Right ICA thrombosis with a free-floating thrombotic mass (25 mm) in the region of the carotid bifurcation, right ischemic stroke
Aldakkan et al. 2017 (18)	Case 1: 69, M Case 2: 85, M	A	Case 1 right (length unspecified) Case 2: left ESP adjacent to the pseudoaneurysm abutting the posterior aspect of the ICA (length unspecified)	Case 1: The AComm and right PComm were absent. When the head was turned to the right, catheter angiography revealed significant right ICA stenosis at the point where the ossified stylohyoid crossed the ICA. The patient experienced a presyncopal episode and left arm weakness in that position during the angiography. Case 2: An 8 mm pseudoaneurysm of the left ICA containing a thrombus and left MCA territory infarct
Radak et al. 2016 (26)	62, F	A	Bilateral	Significant left ICA stenosis >90% and right ICA kinking; stroke 2 years ago, resulting in right-sided weakness
Rama et al. 2020 (15)^*^	64, M	C	Bilateral	Dissection of the right ICA with thrombus and temporoparietal lobe ischemic stroke
Canzoneri et al. 2017 (16)^*^	55, M	C	Bilateral Right: 50.8 mm Left: 51.5 mm	Right ICA dissection and right M1 embolic occlusion
Báez-Martínez et al. 2021 (17)	36, M	A	Bilateral Right: 80 mm Left: 77 mm	Left ICA dissection, left Horner’s syndrome, and no cerebral complication
Rojas et al. 2022 (30)^*^	50, F	C	Left: 66 mm	Abutment of the left ESP on the left ICA, causing compression; no cerebral infarct was noted. Left facial droop, numbness, and weakness in the right lower extremity
Jarrar et al. 2021 (27)	49, M	A	Bilateral Right: 48.5 mm Left: 43.2 mm	Right ICA stenosis >90% without dissection and infarction in the right deep MCA territory

* Conference poster abstracts.

ACA, anterior cerebral artery; AComm, anterior communicating artery; CCA, common carotid artery; CTA, computed tomography angiography; ESP, elongated styloid process; ICA, internal carotid artery; IJV, internal jugular vein; M, Male; MCA, middle cerebral artery; MRI, magnetic resonance imaging; NIHSS, National Institutes of Health Stroke Scale; PComm, posterior communicating artery; F, Female.

Study quality: A: good; B: moderate; C: insufficient; and N/A: not applicable.

Vascular variants of Eagle syndrome are rare. The mean age of onset was observed to be 51 years (range: 15–85 years). The male-to-female ratio was 2:4, indicating a strong male predominance. In most cases (63%), the elongated styloid process (ESP) was bilateral. The ESP length involved in the vascular complications ranged from 31 to 77 mm, with an average length of 48 mm. Vascular complications were mostly represented by internal jugular vein (IVJ) stenosis (28 cases) (4, 5), followed by internal carotid artery (ICA) dissection (15 cases) (1, 2, 6–17). Eight cases of ICA thrombosis were also reported, two of which were caused by the formation of a pseudoaneurysm: one formed directly on the ICA containing the thrombus (18) and the other was due to indirect compression by a giant pseudoaneurysm of the external carotid artery (ECA) on the ICA (19). The mechanism of ICA thrombosis was not specified in the other cases (20–24). A particular case of a patient without an ESP who experienced right ICA thrombosis due to manual compression during sleep was reported (25). Two cases of severe chronic stenosis of the ICA > 90% involving the ESP were also reported (26, 27). Imaging findings typically observed in the ESP cases were mostly focal cortical infarcts due to embolization of the distal branches of the middle cerebral artery. Low carotid flow due to compression by the ESP was found in four cases in the literature, and laminar cortical necrosis as a cerebral complication of this compression was described in only one case. Kazmierski et al. (28) described the case of a patient with a junctional territory infarct between the left middle and anterior cerebral arteries caused by compression and vasospasm of the left ICA by the ESP. This patient had an incomplete circle of Willis, characterized by the absence of the A1 segment of the right anterior cerebral artery and hypoplasia of the P1 segment of the right posterior cerebral artery, which made it impossible for any arterial supply from the right ICA. Kavi and Lahiri (29) reported a similar case of laminar cortical necrosis in the left anterior cerebral artery and middle cerebral artery territories, caused by the compression of the left extracranial ICA by the ESP in a patient with a complete circle of Willis who had low blood pressure after hemodialysis, which made the arterial supply from the right ICA insufficient. Another remarkable case report highlighted the importance of postural involvement in the genesis of low blood flow caused by compression by the ESP (18). The patient experienced a presyncopal episode and left arm weakness when the head was turned to the right. Catheter angiography revealed significant stenosis of the right ICA where the ossified stylohyoid crossed the ICA. The last case was a poster abstract, in which a compression mechanism on the ICA by the ESP was described. However, we did not have enough information to establish this mechanism because the patient experienced bilateral symptoms with only the abutment of the left ESP on the left ICA. In addition, we did not have information about the circle of Willis or the circumstances of the neurological symptoms (30).

## Discussion

Vascular complications of Eagle syndrome are rare and may involve either the IJV, ECA, or ICA. These vascular complications may lead to cerebral ischemia due to either a thromboembolic mechanism or, less frequently, low blood flow, especially as a consequence of insufficient supply from the contralateral ICA in the context of an incomplete circle of Willis or low systemic blood pressure. Laminar cortical necrosis has also been rarely reported in patients with Eagle syndrome. This uncommon observation may be explained by the rarity of the syndrome itself and the specific circumstances required to provoke cerebral damage due to low blood flow, including a postural mechanism favoring the compression of the ICA by the ESP and an insufficient contralateral blood supply. Therefore, in such patients, other potential causes of laminar cortical necrosis must be considered. Laminar cortical necrosis is defined as focal or diffuse necrosis of one or more cortical laminae. The pathophysiology is linked to energy depletion, to which the gray matter is more sensitive than the white matter. It may be caused by a decrease in energy availability, as observed in stroke, hypotension, hypoglycemia, or mitochondrial disease, or by an increase in energy needs, such as in status epilepticus (31). Other factors that create an imbalance between energy needs and availability, causing laminar cortical necrosis, include endocrine, immunological, vascular, metabolic, infectious, toxic, or genetic etiologies.

In conclusion, although Eagle syndrome is rare, it should be considered in the diagnostic work-up of cerebrovascular events. Adequate imaging is thus required to visualize both the vessel lumen and adjacent anatomical structures.
